# CaFe-Based Layered Double Oxides With Superior Iron Alloy Corrosion Inhibition Behaviors in Aggressive Seawater Environment

**DOI:** 10.3389/fchem.2022.813008

**Published:** 2022-02-07

**Authors:** Ji Young Park, Su Been Yoo, Hong-Baek Cho, Han-Seung Lee, Yong-Ho Choa

**Affiliations:** ^1^ Department of Material Science and Chemical Engineering, Hanyang University, Ansan, South Korea; ^2^ Department of Industrial R&D Center, Park Systems Corporation, Suwon-si, South Korea; ^3^ Department of Architectural Engineering, Hanyang University, Ansan, South Korea

**Keywords:** layered double hydroxide, cafe, corrosion, chloride, iron alloy

## Abstract

Reinforced concrete is among the most multifaceted materials used in the construction field. Maintaining the resistance of reinforced concrete to weathering, abrasion, and chemical attack, particularly in aggressive natural conditions such as seawater environments, is challenging. The main factor in the degradation of reinforced-concrete durability is chloride penetration, which accelerates iron alloy corrosion and facilitates structural degradation. In this study, calcium-iron-based layered double hydroxides (CaFe-LDHs) were fabricated at room temperature, followed by structural modulation, and their effectiveness in mitigating iron alloy corrosion due to chloride ions (in 3.5 wt% of NaCl) was investigated. The synthesized CaFe-LDHs with phase transfer notably improved the Cl^−^ removal capacity (Q_max_) to 881.83 mg/g, which is more than three times that reported based on previous studies. The novelty of this research lies in the sophisticated structural and phase transformations of the as-synthesized CaFe-LDHs, determination of crucial factors for chloride ion removal, and suggestion of calcium-iron-based layered double oxide (CaFe-LDO)-based chloride ion removal mechanisms considering chemical and ion-exchange reactions. Moreover, when the phase-transformed LDHs, C-700 LDOs, were applied to inhibit iron alloy corrosion, a noticeable inhibition efficiency of 98.87% was obtained, which was an 11-fold improvement compared to the case of iron alloys without LDOs. We believe this work can provide new insights into the design of CaFe-LDOs for the enhancement of the lifespan of reinforced concrete structures.

## Highlights

• Structural transformation of CaFe-based LDOs in crystallinity and morphology
• Novel reconstruction mechanism of structure-modulated CaFe-LDOs
• Cl- removal capacity of C-700 LDOs, higher than previous reports.
• Inhibition efficiency of 98.87% was obtained comparing with iron alloys without LDOs.
• Potential in the construction industries to enhance the durability of buildings.

## Introduction

Corrosion damage to reinforcement and prestressed steel has been identified as the primary cause of a significant number of structural failures over the past centuries. It also entails a significant cost in terms of the repair, monitoring, and replacement of structures (Berrocal et al., 2016). Reinforced concrete structures are continually exposed to destructive elements that decrease their durability and lifespan. In general, concrete has high alkalinity (pH > 13) and causes iron alloys to develop a passivation layer to prevent corrosion (Ke et al., 2017). The corrosion of reinforcement steel in concrete can only be initiated when the passivation layer is destroyed by anions, particularly chloride ions (Cl^−^). The penetration of chloride into reinforced concrete is a significant threat, specifically for structures near marine environments that are exposed to high concentrations of salts. Such a scenario allows chloride ions to easily penetrate concrete structures through capillary adsorption, hydrostatic pressure, or diffusion into the iron alloys (Pradhan and Bhattacharjee, 2011; Muthulingam and Rao, 2015). Poor concrete quality or insufficient covering of concrete is deleterious to the durability of iron alloys and can lead to substantial economic losses and serious safety disasters (Bassuoni and Nehdi, 2008; Chen et al., 2016). Therefore, the selective and prompt removal of anions that cause deterioration immediately upon their penetration is desirable to maintain the durability and lifespan of concrete structures.

Layered double hydroxides (LDHs), also known as anionic clays, capture anions in aqueous solutions. The structure of LDHs is similar to the regular hexagonal structure of brucite [M(OH)_2_], and their general chemical formula is [M^2+^_1-x_M^3+^_x_(OH)_2_]^x+^[A^n-^_x/n_] ^x−^∙yH_2_O, where M^2+^ (e.g., Ca, Mg, Zn, Co, Ni, Cu) and M^3+^ (e.g., Fe, Al, Cr) are divalent and trivalent metal cations, respectively, and An-is an n-valent anion. Owing to the substitution of M^2+^ with M^3+^ in the brucite-like metallic hydroxide M(OH)_2_, the layered structures have positive charges that are balanced by interlayer anions (An^−^) (Yang et al., 2013; Mishra et al., 2018; Zuo et al., 2019). A unique property of LDHs is that their original structure can be obtained upon rehydration after calcination treatment (Erickson et al., 2005; Santos R. M. M. et al., 2017; Ivánová et al., 2018). In addition, it is well known that various anions can be selectively exchanged without altering the original structure, a feature referred to as the memory effect (Lv et al., 2015). Other advantageous features of LDHs, including their high specific surface area, non-toxicity, and low cost, expand their utilization in the fields of hazardous material adsorption, catalysis, biomedicine, and agriculture (Evans and Duan, 2006; Benício et al., 2015; Lv et al., 2015).

Among the published reports on chloride adsorption with LDHs, a maximum removal capacity of 257 mg/g was reported by Yoon et al. (2014), who compared the adsorption capabilities of various LDHs with different metal hydroxide layers. CaFe-LDHs also show high potential for removing condensed phosphate (Wu et al., 2012). However, the few existing studies are insufficient to understand the adsorption mechanism of CaFe-LDHs, and studies on the anion adsorption capacity are limited.

In this paper, we report the synthesis of structure-modulated calcium-iron-based layered double oxides (CaFe-LDOs) and their outstanding performance in terms of both a high chloride ion removal capacity and iron alloy corrosion protection. To evaluate the effectiveness of CaFe-LDOs in enhancing the durability of concrete, the chloride removal performance and mitigation of iron alloy corrosion by CaFe-LDOs were examined using an aqueous solution of 3.5 wt% NaCl, which corresponds to the concentration of chloride in seawater. In addition, the mechanisms of chloride ion exchange and corrosion protection were investigated.

## Experimental

### Materials and Reagents

CaFe-LDH powders were prepared using calcium nitrate tetrahydrate (Ca(NO_3_)_2_·4H_2_O, 99%), iron nitrate nonahydrate (Fe(NO_3_)_3_·9H_2_O, 98%), sodium hydroxide (NaOH, 99%), and sodium chloride (NaCl, 99.5%), all of which were purchased from Sigma-Aldrich, Inc. and Daejung, Inc. without further purification. Distilled deionized water was used to prepare the standard aqueous ionic solutions.

### Synthesis of CaFe-LDHs

The CaFe-LDHs (pristine LDHs) were prepared via a one-step co-precipitation method (Wu et al., 2012; Gupta et al., 2020; Kong et al., 2021; Romero Ortiz et al., 2021). In particular, the optimized conditions for CaFe-LDH synthesis were determined by varying the Ca^2+^:Fe^3+^ molar ratio in the precursor solutions. First, an aqueous 3 M NaOH solution (300 ml) was rapidly injected at a rate of 300 ml/min into a second solution (300 ml) containing 66.7–80.0 mmol of Ca(NO_3_)_2_·4H_2_O and 33.3–20.0 mmol of Fe(NO_3_)_3_·9H_2_O at room temperature for 1 min to induce supersaturation. This was followed by the aging of the solution for 18 h. The concentration of each salt was balanced according to the Ca^2+^:Fe^3+^ molar ratio, which ranged from 2:1 to 4:1. During this process, the pH was controlled from 12 to 14, and all reactions were performed at room temperature. Upon completion of the reaction, the as-formed precipitates were filtered (200 nm), and the synthesized CaFe-LDHs were washed with excess quantities of ethanol to remove any undesirable nitrate salt residues. The obtained crystals were stored at room temperature in a desiccator to prevent contact with moisture and carbon dioxide in the air.

### Structural Transformation of the CaFe-LDOs

Structural transformations were induced in the as-prepared CaFe-LDHs by thermal treatment. The specimens were heat-treated in a furnace at 400°C (C-400 LDOs) and 700°C (C-700 LDOs) for 3 h in air at a heating rate of 10°C/min.

### Removal Test of Chloride Ion in Aqueous Solution

A chloride solution was prepared by dissolving NaCl in deionized water. The initial concentration of the chloride ions was 200 ppm. Chloride removal was conducted in 200 ml of the aqueous ionic solution after adding 1.89 g of LDHs and LDO under magnetic stirring at 250 rpm.

### Corrosion Protection Test of Iron Alloys

The type of steel used in the corrosion experiments was SS400 with a chemical composition (in wt%) of 0.27 C, 0.90 Mn, 0.20 Cu, 0.05 S, 0.04 Si, and 0.04 P (Fe balance). Iron alloys (1.5 mm) were first sanded and then washed with ethanol. Iron alloy specimens for the corrosion tests were prepared using a simple microstopper coating. The surface on one side of the iron alloys was exposed to perform the corrosion experiment (1 cm × 1 cm), and the remaining area was sealed with a microstopper. An electrolyte solution of 3.5 wt% NaCl, which corresponds to the typical concentration of NaCl in seawater, was prepared. Solution-based chloride adsorption with LDHs and LDOs (pristine LDHs, C-400 LDOs, and C-700 LDOs) and without LDHs and LDOs (bare iron alloys) was monitored. The reaction was initiated by adding 1 g of CaFe-LDH and LDO powder to 250 ml of the corrosion solution. A saturated calomel electrode (SCE) and platinum electrode (PE) were employed as the reference and auxiliary electrodes, respectively.

### Characterization

The solution pH was measured using a FE20 pH meter (Mettler Toledo, United States) equipped with a glass electrode (LE438, Mettler Toledo, United States). Thermogravimetric (TGA) measurements were performed using a Shimadzu TGA-50 thermogravimetric analyzer. The ion concentrations in the liquids after ion exchange were determined by ion chromatography (METROSEP A SUPP 5-250) at a flow rate of 0.7 ml/min with an eluent mixture of 3.2 mM Na_2_CO_3_/1.0 mM NaHCO_3_. Solid samples were characterized by Fourier transform infrared spectroscopy (FT-IR, Thermo Scientific 380 FT-IR) in the range of 4,000 to 500 cm^−1^ with a resolution of 4 cm^−1^; analysis was conducted on a 32 mm disc. X-ray diffraction (XRD) patterns of the solids were acquired using a D/max2200 diffractometer (Rigaku Co., Japan), operating at 40 kV and 100 mA, with Cu Kα radiation (*λ* = 0.1541 nm) in the range of 2θ = 10–80° at a scan rate of 4°/min. The morphology was characterized using a scanning electron microscope (SEM, S-4800, Hitachi Ltd. Japan) equipped with an energy-dispersive X-ray spectroscopy (EDX) detector. The morphology and size of the as-obtained samples were observed by transmission electron microscopy (TEM, JEM-2100F, JEOL, Japan) at an accelerating voltage of 200 kV. For corrosion testing, the anti-corrosive properties and electrical conductivity of the iron alloys were determined by electrochemical measurements, including potentiodynamic polarization (PDP) and electrochemical impedance spectroscopy (EIS) measurements. The EIS measurements were performed by applying a sinusoidal potential perturbation of 10 mV over a frequency range of 100 kHz to 100 MHz. The obtained EIS spectra were analyzed with an equivalent circuit using the Zman software. All electrochemical measurements were performed under quiescent conditions. No attempt was made to aerate or de-aerate the test solutions.

## Results and Discussion

### Characterization of the Synthesized CaFe-LDHs

CaFe-LDHs with nitrate $\left({\text{NO}}_{3}^{-}\right)$ anions intercalated in the interlayer region were synthesized by varying the pH (from 12 to 14) and Ca^2+^:Fe^3+^ molar ratio (from 2:1 to 4:1). XRD patterns of the as-synthesized LDHs are displayed in Supplementary Figure S1. The LDHs prepared at a 2:1 M ratio of Ca^2+^:Fe^3+^ resulted in CaFe-LDHs with a crystal structure corresponding to hexagonal Ca_2_Fe(OH)_6_(NO_3_)∙2H_2_O (JCPDS No. 48-65), as shown in Supplementary Figure S1A. Such a structure is intercalated with NO_3_^−^ anions and crystalline water, and the diffraction peaks are located at 2θ values of approximately 10.12°, 20.40°, and 30.28 °for the (001), (002), and (110) planes, respectively. An additional impurity phase, CaO, first appeared when the Ca^2+^:Fe^3+^ molar ratio reached 3:1. With a further increase in the Ca^2+^:Fe^3+^ molar ratio to 4:1, both CaO and Ca(OH)_2_ phases appeared (Supplementary Figure S1B). The calculated basal spacing values were 8.73, 8.63, and 8.57 Å for Ca^2+^:Fe^3+^ molar ratios of 2:1, 3:1, and 4:1, respectively. The structural parameters and crystallite sizes of the Ca^2+^:Fe^3+^ LDH samples are listed in Table 1. The mean crystallite size (D) was estimated according to the Scherrer equation as (1) (Dorn, 1953):

$$D=\frac{k\lambda }{\beta \cos\theta }$$ (1)

where K is the shape factor, *λ* is the X-ray wavelength, β is the full width at half maximum for a given diffraction peak after correcting for instrumental broadening, and θ is the Bragg angle of reflection.

**TABLE 1:** Structural parameters from XRD analysis of CaFe-LDHs with different Ca^2+^:Fe^3+^ molar ratios.

Ca^2+^:Fe^3+^	*d*_(001)_ (Å)	*d*_(002)_ (Å)	*d*_(110)_ (Å)	Lattice parameters (Å)	Crystallite size (nm)	
					(001)	(110)
2:1	8.73	4.35	2.95	8.61	28.45	29.36
3:1	8.63	4.33	2.94	8.62	15.93	37.37
4:1	8.57	4.32	2.95	8.65	14.23	34.25

As shown in Supplementary Figure S1B, when the co-precipitation reaction was performed at a pH less than 13, no CaFe-LDH crystallites were formed. With an increase in the initial pH to 14, CaFe-LDHs interacted with the (OH)^-^ anion, exhibiting a lower crystallinity owing to an increase in the formation of impurities, including Ca(OH)_2_ and Fe(OH)_3_. The obtained results demonstrate that well-crystallized single-phase CaFe-LDHs can be synthesized at a pH of 13.

### Structure Transformation of CaFe-LDOs With Thermal Decomposition

**FIGURE 1:**
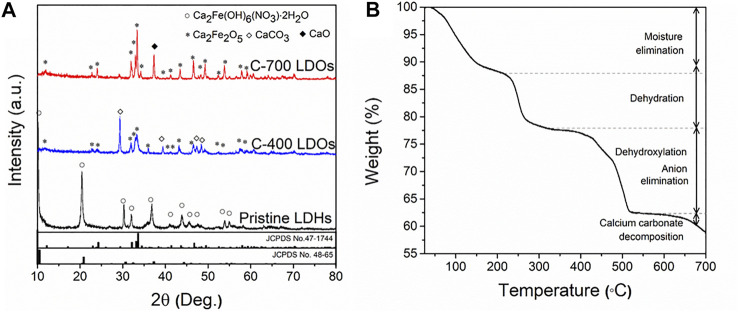
**(A)** XRD patterns of Pristine LDHs, C-400 LDOs, and C-700 LDOs with different structural transformations and **(B)** TGA spectrum.

Figure 1A shows the XRD patterns after the thermal treatment. In the CaFe-LDHs calcined at different temperatures, remarkable phase changes were observed. Specifically, the original structure of pristine LDHs was completely destroyed and transformed into Ca_2_Fe_2_O_5_ (JCPDS No. 47-1744) with an orthorhombic structure. A CaCO_3_ (JCPDS No. 86-2339) impurity phase, indexed to a rhombohedral structure with a main diffraction peak at 2θ = 29.36°, was subsequently generated. As evident from the XRD patterns in Figure 1A, the CaCO_3_ phase only appeared when pristine LDHs were treated at 400°C under ambient conditions. However, when the C-400-LDO specimen was treated in Ar, no CaCO_3_ phase was formed (Supplementary Figure S2). This finding suggests that CaCO_3_ formation is related to CO_2_ dissolution from the air during the calcination process. As the calcination temperature increased, the CaCO_3_ phase disappeared, and the crystallization of the CaO phase increased. When the temperature exceeded 550°C, decomposition occurred (Babou-Kammoe et al., 2012; Yamaguchi et al., 2015). The disappearance of CaCO_3_ can be explained by the following reaction:

$$CaCO_{3\left(s\right)}\to CaO_{\left(s\right)}+CO_{2\left(g\right)}$$ (2)

TGA data were acquired to explore the thermal decomposition of the synthesized CaFe-LDHs. The results are shown in Figure 1B. Four major weight loss stages were observed. The first weight loss stage (11.7 wt%) occurred from room temperature to 200°C and corresponds to the loss of weakly adsorbed water molecules on the crystallite surface. During the second stage of weight loss (9.9 wt%) between 200 and 300°C, the intercalated crystallized water between the cationic layers was removed. The third stage of weight loss (15.86 wt%), which occurred from 300 to 550°C, was associated with the thermal decomposition of hydroxyl (OH^−^) groups anchored to transition metal oxides and anionic (NO_3_^−^) species (Mahjoubi et al., 2017). Finally, the last stage of weight loss (3.7 wt%), which appeared at temperatures above 550°C, was attributed to the decomposition of calcium carbonate (CaCO_3_), as displayed in Supplementary Figure S3.

The FT-IR spectra of the CaFe-LDH samples were analyzed to identify structural transformations via comparisons of chemical bonding involving various functional groups (Supplementary Figure S4). The characteristic peaks appearing at 1,640 cm^−1^ are assigned to the H-O-H bending vibrations of interlayer molecular water, whereas the characteristic sharp absorption band around 1,350 cm^−1^ is associated with the antisymmetric stretching mode of NO_3_^−^ in the interlayer (Supplementary Figure S4B). The intense broad bands observed at approximately 3,585 cm^−1^ are associated with the stretching vibrations of the structural (OH^−^) groups in Ca(OH)_2_ and Fe(OH)_2_ (Supplementary Figure S4C). The peaks at 749 and 580 cm^−1^ are attributed to the stretching vibrations of either Ca-O or Fe-O (Metal-O) in the lattice, as shown in Supplementary Figure S4D (Wu et al., 2012; Mahjoubi et al., 2017). The band at 1,640 cm^−1^ disappeared completely after calcination in the case of C-400 LDO because the crystallized water molecules were removed. In addition, the gradual decrease in the NO_3_^−^ stretching vibration intensity at 1,350 cm^−1^ implies the decomposition of NO_3_^−^ anions due to an increase in the calcination temperature; complete decomposition occurred in the C-700 LDO sample. A noticeable decrease in peaks corresponding to the OH^−^ group in the case of C-400 LDO implies that the OH^−^ species of the Ca(OH)_2_ and Fe(OH)_2_ cationic layers were decomposed when the thermal treatment temperature exceeded 400°C. The subsequent increase in the intensity of the metal-O peaks at 749 and 580 cm^−1^ indicates that the structure of the pristine LDHs was transformed into that of the respective metal oxides. Peaks corresponding to commercial CaCO_3_ appeared at 1,394 cm^−1^ (υ^3^ asymmetric CO_3_^2−^) and 871 cm^−1^ (υ^2^ asymmetric CO_3_^2−^) (Butto et al., 2018). The intensity of these bands decreased with increasing calcination temperature. Thus, according to the XRD and TGA findings, a structural transformation occurred in the C-400 LDO specimen.

**FIGURE 2:**
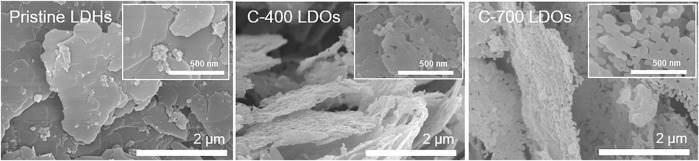
SEM images of **(A)** Pristine LDHs, **(B)** C-400 LDOs and **(C)** C-700 LDOs.

Figure 2 shows SEM images of the CaFe-LDHs for different thermal treatment conditions. A comparison of the CaFe-LDHs with pristine LDHs revaled transformed morphology in C-400 LDOs. Such a phenomenon is influenced by the destruction of anion layers with the removal of crystalline water molecules. The C-700 LDOs specimen presented a morphology consisting primarily of individual spherical particles.

**FIGURE 3:**
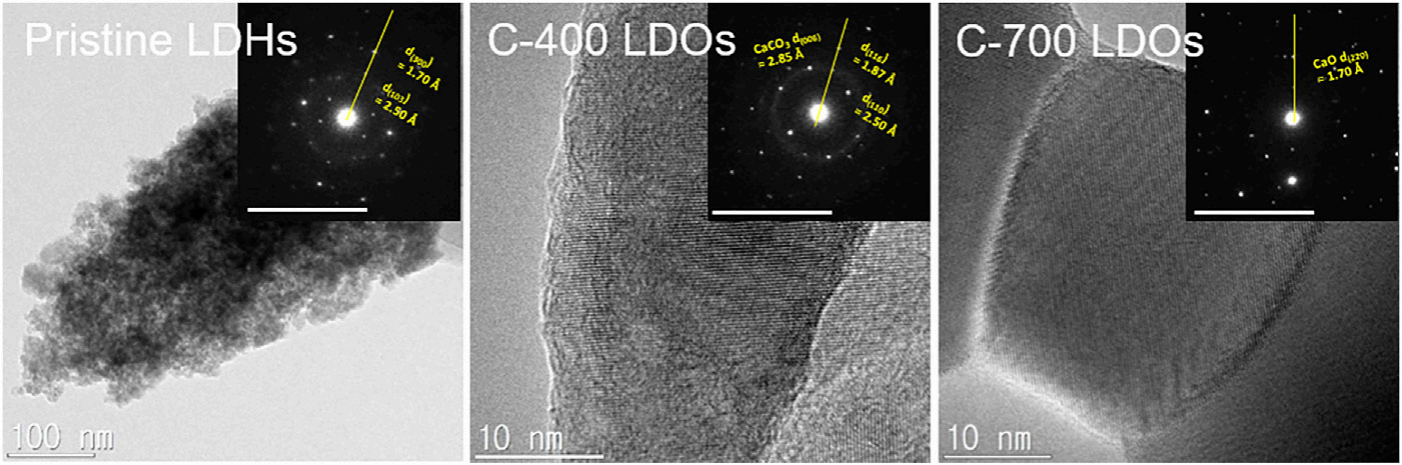
TEM images and SAED patterns of **(A)** Pristine LDHs, **(B)** C-400 LDOs, and **(D)** C-700 LDOs.

Figure 3 displays the TEM images and the associated selected area electron diffraction (SAED) patterns obtained for the CaFe-LDH samples. The pristine LDHs showed the typical crystallographic layered structure of CaFe-LDHs, including (300) and (103) planes with lattice constants of 1.70 Å and 2.50 Å, respectively. For C-400 LDOs, the presence of the (006), (110), and (116) planes corresponding to rhombohedral CaCO_3_ was detected after thermal treatment at 400°C. In the case of calcination at 700°C, only the CaO phase was identified in the XRD pattern (Figure 1A). Here, CaO is a unique by-product formed as a result of thermal treatment. The obtained SAED patterns confirm the occurrence of gradual structural and crystallographic transformations as a function of temperature from 400 to 700°C.

### Adsorption Performance of Chloride in Aqueous Solution

**FIGURE 4:**
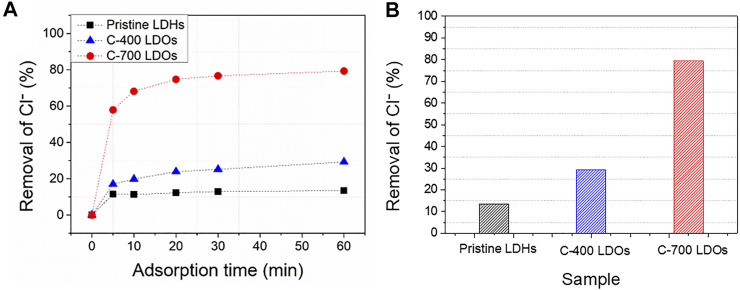
Ion chromatography of CaFe-LDHs chloride removal (%) at **(A)** different adsorption times and **(B)** a fixed adsorption time of 60 min.

Experiments were conducted to investigate the removal of chloride ions, which may shorten the lifespan of building structures. Figures 4A,B show the results obtained for the removal of chloride at different adsorption times and a fixed ion adsorption time of 60 min, respectively. The percentage removal efficiency was estimated according to Equation (3).

Removal (%)=C0−CeC0×100 (3)

where $C_{0}$ and $C_{e}$ are the initial and equilibrium anion concentrations (mg/L), respectively.

As shown in Figure 4A, the removal percentages of pristine LDHs, C-400 LDOs, and C-700 LDOs were 11.49, 16.98, and 57.90%, respectively, for an adsorption time of 5 min at the initial stage of the reaction. The amount of adsorbed chloride increased rapidly over 5 min, with a gradual increase up to 60 min before reaching steady-state equilibrium. The removal percentages for a 60 min adsorption time were 13.41% for pristine LDHs, 29.25% for C-400 LDOs, and 79.62% for C-700 LDOs. Based on the obtained findings, the removal efficiency with C-700 LDOs was almost six times higher than that of pristine LDHs, suggesting outstanding performance in terms of chloride removal.

The FT-IR spectra of the sample upon the adsorption of chloride ions are illustrated in Supplementary Figure S5. The characteristic sharp absorption band at approximately 1,360 cm^−1^ is associated with the antisymmetric stretching mode of chloride in the interlayer. In addition, in the cases of C-400 LDOs and C-700 LDOs without anion adsorption, typical H-O-H peaks corresponding to bending vibrations (1,640 cm^−1^) and stretching vibrations (3,585 cm^−1^) induced by (OH)^-^ groups were removed (see comment in Supplementary Figure S4). However, the adsorption peaks reappeared after chloride adsorption due to the recovery of (OH)^-^ groups and crystalline water molecules during the reconstruction process in an aqueous atmosphere. Hence, the results provide evidence for both the reconstruction processes and the successful adsorption of chloride ions by CaFe-LDOs.

**FIGURE 5:**
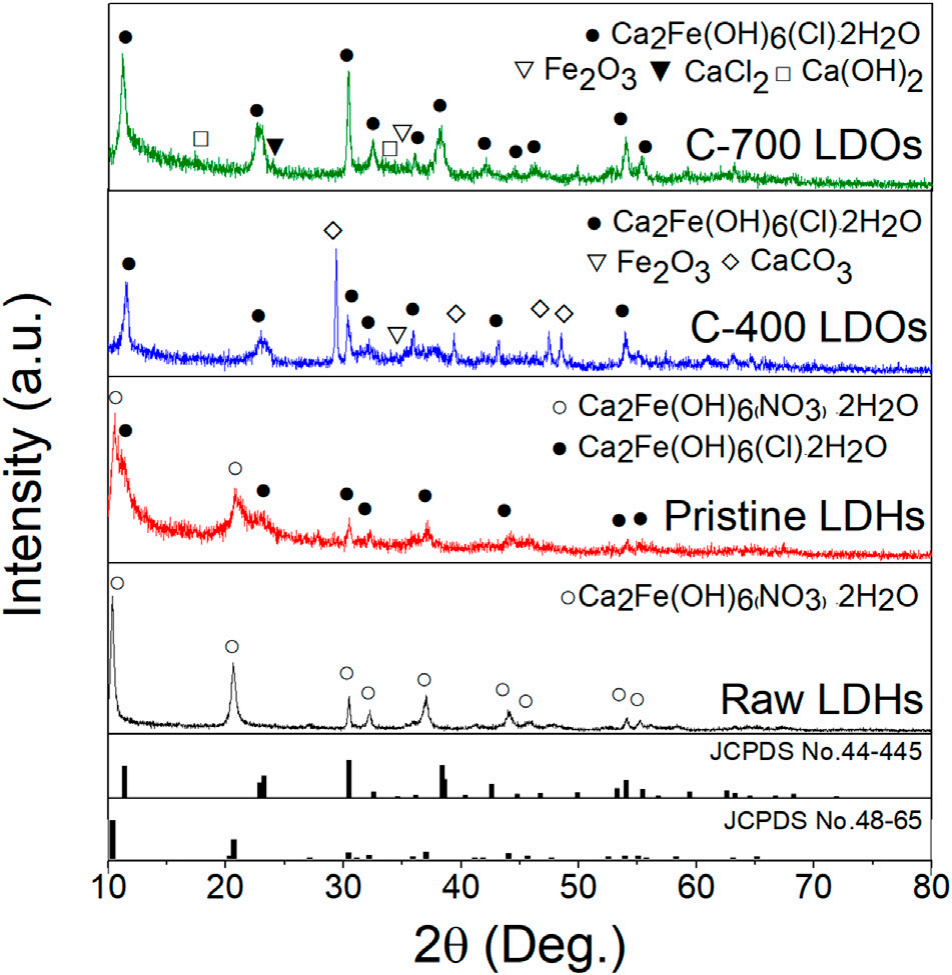
XRD patterns of before the chloride adsorption (Raw LDHs), Pristine LDHs, C-400 LDOs, and C-700 LDOs after chloride adsorption.

The reconstructed CaFe-LDHs and LDOs after 60 min of chloride adsorption were characterized by XRD. The results are shown in Figure 5. Characteristic peaks that were typical of CaFe-LDHs were indexed to the rhombohedral structure for all LDH samples, including those from the (006) and (0012) planes after the adsorption of chloride ions. Upon chloride adsorption, the XRD patterns of the crystalline LDHs corresponded to a structure with a chemical formula Ca_2_Fe(OH)_6_(Cl)∙2H_2_O (JCPDS No.44-445). The crystallinity of the CaFe-LDHs simultaneously decreased with anion adsorption due to the reconstruction phenomenon (i.e., memory effect). Furthermore, the basal spacing increased, and the 2θ angle associated with the (006) XRD peak increased from 10.36° (NO_3_^−^) to 11.32° (chloride) because the ionic radius of the chloride ion (1.75 Å) is smaller than that of NO_3_^−^ (1.79 Å). The chloride ion was partially substituted with NO_3_^−^ present in the interlayer, which led to the formation of two types of interstratified pristine LDHs. The pre-adsorption data are shown in Figure 1A. The XRD pattern of the post-adsorption C-400 LDOs also included secondary crystalline phases of CaCO_3_ (2θ = 29.36°) even after particle reconstruction because CaCO_3_ is a basic salt with very low solubility. Therefore, the layered CaFe- LDOs reconstructed via refolding in aqueous solution could exchange chloride anions due to the reconstruction of Ca_2_Fe_2_O_5_ as follows (4):

$$Ca_{2}Fe_{2}O_{5}+2CaCO_{3}+Cl^{-}+5.5H_{2}O\to \;Ca_{2}Fe{\left(OH\right)}_{6}\left(\text{Cl}\right)\cdot 2H_{2}O+0.5Fe_{2}O_{3}+2CaCO_{3}+OH^{-}$$ (4)

In particular, the CaFe-LDOs prepared after thermal treatment at 700°C showed the pure crystalline structure of CaFe-LDHs with individual interlayer anions of Cl^−^, Ca(OH)_2_, and CaCl_2_. This was because the secondary crystalline phase of CaO (see Figure 1A) after anion adsorption was transformed to Ca(OH)_2_ via hydrolysis in solution. The results suggest adsorption from partial Ca(OH)_2_ phases to CaCl_2_. Therefore, the C-700 LDOs sample undergoes the following chemical reaction when reconstructed in water containing chloride (5):

$$Ca_{2}Fe_{2}O_{5}+2CaO+4Cl^{-}+7.5H_{2}O\to \;Ca_{2}Fe{\left(OH\right)}_{6}\left(\text{Cl}\right)\cdot 2H_{2}O+0.5Fe_{2}O_{3}+0.5Ca{\left(OH\right)}_{2}+1.5CaCl_{2}+4OH^{-}$$ (5)

This mechanism was induced by the following factors: 1) anion capture by reconstruction, 2) capture by chemical reaction with metal oxide, and 3) physical adsorption. The C-700 LDOs can capture 4 mol of Cl^−^ (see Eq. (5)). Here, 1 mol of Cl^−^ was captured by intercalation between cationic layers during the refolding process, and additional 3 mol of Cl^−^ were captured by chemical reaction with CaO, which was generated during thermal treatment at 700°C. This metal oxide moiety, initiated by pristine-LDHs, also captures Cl^−^ by chemical reactions other than capture by the refolding process, which cannot be realized even after the calcination of typical LDHs (Wu et al., 2012; Halajnia et al., 2013; Zhou et al., 2015). Supplementary Figure S6 shows SEM images of the reconstructed pristine LDHs, C-400 LDOs, and C-700 LDOs before (top) and after anion adsorption (bottom). Although no significant changes were observed for the reconstructed LDHs, differences in morphology between the pristine LDHs and C-700 LDOs are evident. Although the morphologies of pristine LDHs before and after anion adsorption appear to be similar, the post-anion adsorption C-700 LDOs exhibit larger plate-like particles aggregated into flower-like shapes. Such a microstructure may be attributed to the change in the crystallinity of the remaining Ca(OH)_2_ phases, as expressed by Eq. 9. The removal of chloride ions by CaFe-LDOs particles could be influenced by structural tuning. In particular, the CaFe-LDOs exhibited different efficiencies (%) for anion removal, which could be enhanced by using CaFe-LDOs rather than pristine LDHs.

### Adsorption Kinetics and Equilibrium Sorption Measurements and Modeling

**FIGURE 6:**
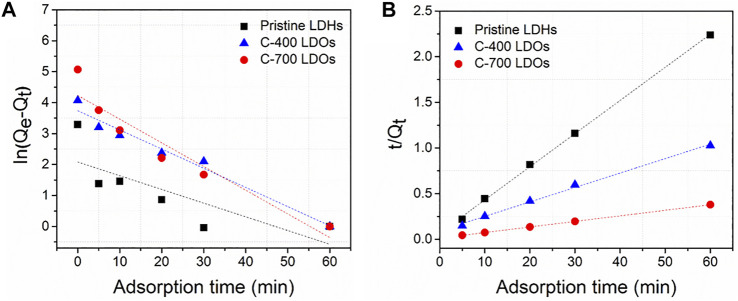
**(A)** Pseudo-first-order and **(B)** pseudo-second-order kinetic plots for the sorption of chloride on Pristine LDHs, C-400 LDOs and C-700 LDOs.

To investigate the difference in anion adsorption by the CaFe-LDHs and LDOs, the adsorption kinetics of chloride ions onto the CaFe-LDHs and LDOs were fitted to a linearized form of pseudo-first-order (Eq. 6) and pseudo-second-order (Eq. 7) models based on experimental data (Calisto et al., 2019; Rathee et al., 2019):

$$\ln\left(Q_{e}-Q_{t}\right)=\ln Q_{e}-k_{1}t$$ (6)

$$\frac{t}{Q_{t}}=\frac{1}{k_{2}Q_{e}^{2}}+\frac{t}{Q_{e}}$$ (7)

where $Q_{e}$ and $Q_{t}$ denote the amounts of anions adsorbed (mg/g) per unit of LDHs and LDOs at equilibrium and at a specified time *t* (min), respectively; and $k_{1}$ and $k_{2}$ denote the pseudo-first-order and pseudo-second-order rate constants, respectively. Pseudo-first-order (Figure 6A) and pseudo-second-order (Figure 6B) plots for the sorption of anions by CaFe-LDHs and LDOs as a function of adsorption time are depicted in Figure 6. The specific model parameters obtained for chloride ion concentrations are listed in Table 2. The correlation coefficients (*R* ^2^) were compared to determine the adequate kinetic model for the quantitative evaluation. Among the experimental data, the chloride ion adsorption kinetics were only slightly fitted to the pseudo-second-order plot and did not follow the pseudo-first-order plot. In contrast, well-fit kinetics were observed when the data were evaluated using a pseudo-second-order plot. As shown in Table 2, the high correlation coefficient values (*R* ^2^) obtained for all the LDH and LDO adsorbent samples confirm that the pseudo-second-order model describing the chloride ion kinetics is well-fitted when compared to the pseudo-first-order model (Zaghouane-Boudiaf et al., 2011). Therefore, chemisorption, which typically involves electrostatic attraction and chemical bonding between the adsorbate and adsorbent, is responsible for the immobilization of chloride ions on CaFe-LDHs and LDOs.

**TABLE 2:** Coefficients of pseudo-first-order and pseudo-second-order parameters for chloride adsorption by pristine LDHs, C- 400 LDOs, and C-700 LDOs.

LDHs	Pseudo-first-order kinetic parameters			Pseudo-second-order kinetic parameters		
	*K*_1_ (min^−1^)	*Q*_*e*_ (ppm)	*R* ^2^	*K*_2_ (ppm^−1^min^−1^)	*Q*_*e*_ (ppm)	*R* ^2^
Pristine LDHs	0.0443	13.1113	0.5374	0.0363	27.5460	0.9991
C-400 LDHs	0.0620	41.3215	0.9663	0.0158	63.2302	0.9950
C-700 LDHs	0.0765	93.5092	0.8970	0.0061	164.0372	1

**FIGURE 7:**
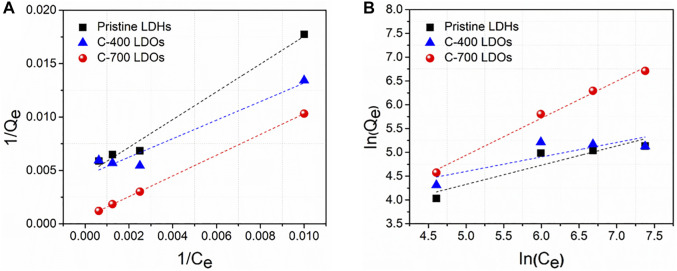
**(A)** Langmuir and **(B)** Freundlich plots for the sorption of chloride onto Pristine LDHs, C-400 LDOs, and C-700 LDOs.

The aim of adsorption isotherms is to correlate the adsorbate concentration in the solution with the adsorbed content of metal ions at the adsorbate-to-adsorbent interface. Langmuir (Figure 7A) and Freundlich (Figure 7B) isotherm models are crucial for the design of sorption systems. The adsorption of chloride ions onto CaFe-LDH and LDO adsorbents was investigated, and the applicability of the aforementioned isotherms was examined. The Langmuir model assumes a homogenous surface via monolayer adsorption between the adsorbed ions, and the Langmuir isotherm represents the equilibrium distribution of sorbate between the solid and liquid phases. This isotherm is often expressed as Equation 8 (Yan et al., 2016; Mu’azu et al., 2018):

$$Q_{e}=\frac{Q_{max}K_{L}C_{e}}{1+K_{L}C_{e}}$$ (8)

where $C_{e}$ (mg∙L^−1^) denotes the concentration of sorbate at equilibrium, $Q_{e}$ (mg ∙g^−1^) denotes the amount of sorbate per mass of sorbent at equilibrium, $K_{L}$ (L∙mg^−1^) represents the equilibrium constant related to the sorption energy between the sorbate and sorbent, and $Q_{max}$ denotes the limiting amount of sorbate that can be taken per mass of sorbent.

The Freundlich equation can be utilized to estimate the sorption intensity of the sorbent toward the sorbate, and is expressed by Equation 9 (Peng et al., 2015; Santos R. M. M.d. et al., 2017):

$$Q_{e}=K_{F}C_{e}^{1/n}$$ (9)

where $Q_{e}$ (mg∙g^−1^) denotes the amount of adsorbed anions, $C_{e}$ denotes the equilibrium concentration, and $K_{F}$ and n are constants incorporating all parameters affecting the sorption process, such as the sorption capacity and intensity. The isotherm constants in Equations 8, 9, as well as the correlation coefficients (*R* ^2^), are listed in Table 3. Based on all the *R* ^2^ values of the LDH and LDO samples and the maximum capacities, the equilibrium data fit the Langmuir isotherm with a high correlation coefficient, indicating monolayer sorption of chloride ions onto CaFe-LDHs and LDOs. In particular, the Langmuir parameters are Qmax = 78.97 mg/g for pristine LDHs, Qmax = 110.23 mg/g for C-400 LDOs, and Qmax = 881.83 mg/g for C-700 LDOs. The removal of chloride anions by different LDHs and LDOs has been studied in recent years, and some reports have provided the uptake capacity for chloride anions. Table 4 shows that the uptake capacity obtained in this study was larger than that reported in previous research.

**TABLE 3:** Langmuir and Freundlich isotherm parameters for chloride adsorption by pristine LDHs, C- 400 LDOs, and C-700 LDOs.

LDHs	Langmuir isotherm parameters			Freundlich isotherm parameters		
	*K*_*L*_ (L∙mg^−1^)	*Q*_*max*_ (mg)	*R* ^2^	*K*_*F*_ (L∙mg^−1^)	*n*	*R* ^2^
Pristine LDHs	0.0108	78.9704	0.9793	3.9992	1.8914	0.7935
C-400 LDHs	0.0043	110.2291	0.9203	17.9987	3.0321	0.5468
C-700 LDHs	0.0006	881.8342	0.9999	3.1183	1.3050	0.9882

**TABLE 4:** Comparison of LDHs removal capacity for chloride anions obtained in this work and in previous reports.

LDH sorbent	Removal capacity (mg/g)	Reference
Pristine LDHs	78.9704	This work
C-400 LDHs	110.2291	
C-700 LDHs	881.8342	
MgAl LDHs	257.00 mg/g	*Materials Chemistry and Physics*, 2014, 145, 376e386
MgAl LDHs	168.00 mg/g	*Water research*, 2006, 40, 735–743
MgAl LDHs	149.50 mg/g	*Desalination and Water Treatment*, 2011, 36, 50–56
MgAl LDHs	128.20 mg/g	*Corrosion Science*, 2019, 152, 120–129
MgAl LDHs	122.20 mg/g	*Chemosphere*, 2018, 209, 721–729
MgAl LDHs	115.50 mg/g	*Journal of Hazardous Materials*, 2015, 300, 475–482
CaAl LDHs	105.90 mg/g	*Desalination*, 2020, 474, 114,186
CaAl LDHs	105.00 mg/g	*Construction and Building Materials*, 2015, 93, 1,051–1,058
MgAl LDHs	33.54 mg/g	*Applied Clay Science*, 2020, 187, 105,495

### Corrosion of Iron Alloys in Aqueous Solution With and Without LDHs

**FIGURE 8:**
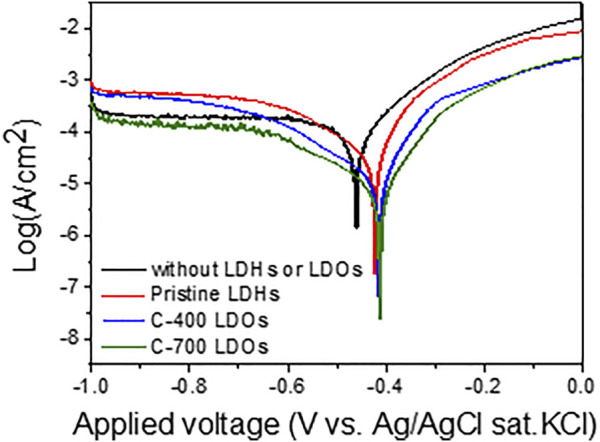
Potentiodynamic polarization (PDP) curves obtained in a 3.5 wt% NaCl solution without LDHs or LDOs and with Pristine LDHs, C-400 LDOs, and C-700 LDOs.

To study the anti-corrosion effects of CaFe-LDHs and LDOs on iron alloys from destructive chloride anions, potentiodynamic measurements of the exponential dependence of the current on voltage deviations were conducted in an aqueous 3.5 wt% NaCl solution; the results are depicted in Figure 8. The variations in the electrochemical kinetic parameters, such as the corrosion current (I_corr_) and corrosion rate (CR), were determined from the curves using the extrapolation method (as plotted in Figure 8 and supplemented by Table 5).

**TABLE 5:** Electrochemical polarization parameters and calculated corrosion rates (mm/year) in a 3.5 wt% NaCl solution with and without LDHs or LDOs.

Sample	Parameter *E*_*corr*_ (mV)	*I*_*corr*_ (µA)	*Β*_*a*_ (mV dec^−1^)	*Β*_*c*_ (mV dec^−1^)	*CR* (mm/year)
Without LDHs	−461.21	206.04	188.11	350.14	2390.69 × 10^–3^
Pristine LDHs	−423.175	34.978	64.61	190.67	405.85 × 10^–3^
C-400 LDHs	−415.179	12.225	71.23	179.18	141.85 × 10^–3^
C-700 LDHs	−411.107	7.695	80.62	174.4	89.28 × 10^–3^

The corrosion rates (mm year^−1^) obtained from the polarization were calculated using Equation 10:

$$CR=\frac{I_{corr}\times K\times EW}{\rho \times A}$$ (10)

Here, EW denotes the equivalent weight of Fe (27 g), K represents the corrosion rate constant (3,272 mm year^−1^A^−1^ cm^−1^), denotes the density of Fe (7.874 gcm^−3^), and A (cm^2^) denotes the surface area of the electrode.

**FIGURE 9:**
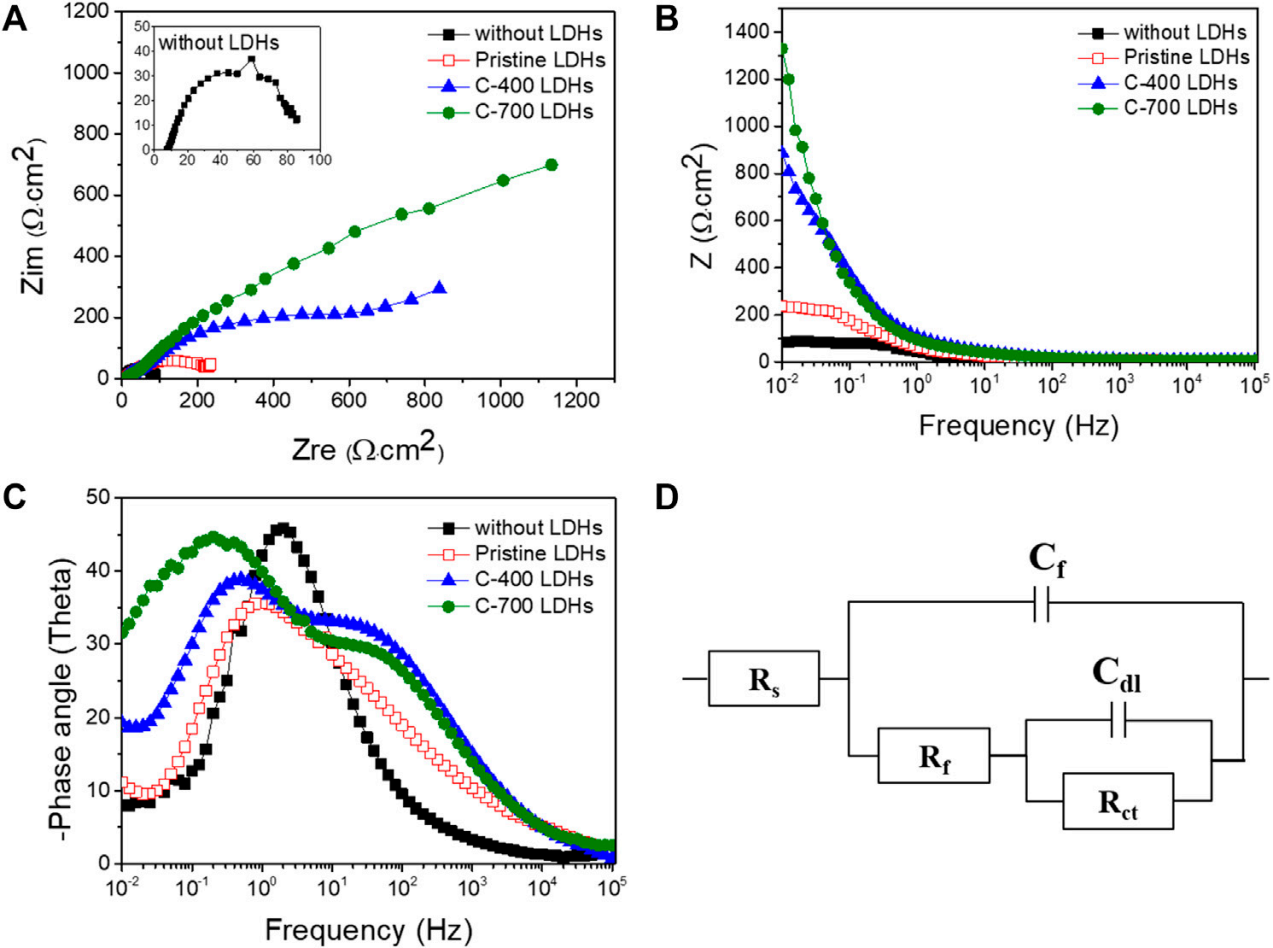
**(A)** Nyquist plots, **(B)** Bode plots, and **(C)** phase angle plots collected at corrosion potentials in a 3.5 wt% NaCl solution without LDHs or LDOs and with Pristine LDHs, C-400 LDOs, and C-700 LDOs. **(D)** Equivalent electrical circuit (EEC) used to fit the electrochemical impedance data.

The CR for iron alloys with the application of C-700 LDHs was 89.28 × 10^–3^ mm/year, whereas the CR in the case without LDHs and LDOs was 2390.69 × 10^–3^ mm/year. These findings indicate that when iron alloys are placed in a severe atmosphere with a high concentration of chlorides in solution, the speed of corrosion is accelerated to a maximum of 26.7 times over a year unless the iron alloys are not protected. As can be seen from the results of the Nyquist plot for iron alloys upon the application of CaFe-LDHs and LDOs (Figure 9A), the mitigation of corrosion led to a clear increase in the diameter of the capacitive loop. The diameter of the semicircle was also significantly enlarged for the protective LDH and LDO samples containing more charge-transfer resistance, as confirmed by the Bode plots in Figure 9B. To quantitatively determine the effects of various factors, including the addition of CaFe-LDHs and LDOs, immersion time, and chloride content, a numerical simulation must be employed to fit the obtained EIS results. Because the degree of chloride removal from the solution containing the iron alloy specimen is an indicator of corrosion protection, the equivalent circuit model constants in Figure 9D were applied. In the equivalent circuit model, iron alloys are considered to have a porous structure and exhibit capacitive behavior (Hou et al., 2019; Imanieh and Afshar, 2019; Liu et al., 2019; Xu et al., 2020). The relevant parameters are listed in Table 6, where R_s_ denotes the electrolyte resistance, R_f_ denotes the electrolyte resistance inside the pores, C_f_ represents the film capacitance, R_ct_ represents the charge transfer resistance of the electrochemical processes occurring inside the pores, and C_dl_ denotes the double-layer capacitance. The C_dl_ value for pristine LDHs was 3.26 × 10^–3^ (µF·cm^−2^), and this parameter generally decreased with higher-temperature heat treatment; the C_dl_ values of C-400 LDOs and C-700 LDOs were 2.65 × 10^–3^ (µF·cm^−2^) and 2.45 × 10^–3^ (µF·cm^−2^), respectively. This phenomenon can be explained according to the Helmholtz model, where C_dl_ is expressed as follows (11) [15]:

$$C_{dl}=\frac{\varepsilon_{r}\varepsilon_{0}}{d}S$$ (11)

where S denotes the exposed surface area of the steel specimen, d represents the thickness of the double layer, ε_r_ denotes the dielectric constant of the electrolyte inside the capacitor, and ε_0_ denotes the vacuum dielectric constant. Here, an increase in C_dl_ indicates an increase in the exposed surface area of the bare iron alloys by corrosion. The addition of LDHs and LDOs decreases the aggressive chlorination reaction (see Equations 1–4 and corrosion of the steel specimen is significantly alleviated. Hence, the reduction in the C_dl_ value is mainly attributed to a decrease in the exposed iron alloy surface area due to the presence of the LDHs. It is well known that the corrosion rate of steel is inversely proportional to the R_ct_ value. In a solution without LDHs, the Rct value was 39.57 Ω cm^2^. However, the R_ct_ value with pristine LDHs was 159.62 Ω cm^2^, and this parameter gradually increased to a maximum of 3,520.01 Ω cm^2^ for C-700 LDOs. To more clearly illustrate the corrosion mitigation effects of CaFe-LDHs and LDOs, the inhibition efficiency (IE%) of the CaFe-LDHs and LDOs was calculated according to the following Equation 12:

$$IE\left(\%\right)=\frac{R_{ct,with\;LDH}-R_{ct,without\;LDH}}{R_{ct,with\;LDH}}$$ (12)

where R_ct_, with LDHs and LDOs, and R_ct_, without LDHs, represent the charge transfer resistances of the steel specimens in the presence and absence of CaFe-LDHs and LDOs, respectively. From Table 6, it is evident that the solution containing both the iron alloy specimen and CaFe-LDHs and LDOs showed an inhibitory effect, whereas no effect was observed in the test solution without LDHs. In particular, the highest inhibition efficiency of 98.87% was achieved with the C-700 LDOs.

**TABLE 6:** Impedance parameters and calculated inhibition efficiencies (IE%) in a 3.5 wt% NaCl solution with and without LDHs or LDOs.

Sample	Parameter *R*_*s*_ (Ω·cm^2^)	*C*_*f*_ (µF·cm^−2^)	*R*_*f*_ (Ω·cm^2^)	*C*_*dl*_ (µF·cm^−2^)	*R*_*ct*_ (Ω·cm^2^)	IE (%)
Without LDHs	7.64	3.23 × 10^–3^	9.92	8.04 × 10^–3^	39.57	—
Pristine LDHs	8.25	0.29 × 10^–3^	22.11	3.26 × 10^–3^	159.62	75.19
C-400 LDHs	9.41	0.21 × 10^–3^	662.07	2.65 × 10^–3^	663.12	94.03
C-700 LDHs	25.15	0.17 × 10^–3^	3,519.89	2.45 × 10^–3^	3,520.01	98.87

## Conclusion

Structure-modulated CaFe-LDHs were fabricated at room temperature using a modified co-precipitation method. A high Cl^−^ adsorption capability of 881.83 mg/g was achieved with C-700 LDOs (LDHs heat treated at 700°C). The chloride removal capability exceeded three times the maximum performance in previous studies (257 mg/g) on chloride removal by LDHs. It was also confirmed that the kinetics were well fitted by a pseudo-second-order model, suggesting dominant chemical adsorption on CaFe-LDHs and LDOs. The Langmuir isotherm models confirmed that the LDHs and LDOs effectively removed the target anions, primarily via monolayer sorption. Moreover, it was also confirmed that crystalline CaO was formed as a C-700 LDO moiety during the heat treatment of pristine CaFe-LDHs, and this phase served as another source to enhance the Cl^−^ removal by undergoing a chemical reaction. In particular, the highest inhibition efficiency of 98.87% was achieved with C-700 LDOs compared to iron alloys without LDHs. Based on the results obtained in this study, CaFe-LDOs demonstrate considerable potential for use as concrete fillers or additives in the construction industry to enhance the durability/sustainability of iron alloy reinforced buildings.

## Data Availability

The original contributions presented in the study are included in the article/Supplementary Material, further inquiries can be directed to the corresponding author.
