# Supplementary figures and images for: CaFe-Based Layered Double Oxides With Superior Iron Alloy Corrosion Inhibition Behaviors in Aggressive Seawater Environment
Source: Front Chem. 2022 Feb 7;10:813008. doi: 10.3389/fchem.2022.813008 (PMC8858811; doi:10.3389/fchem.2022.813008)

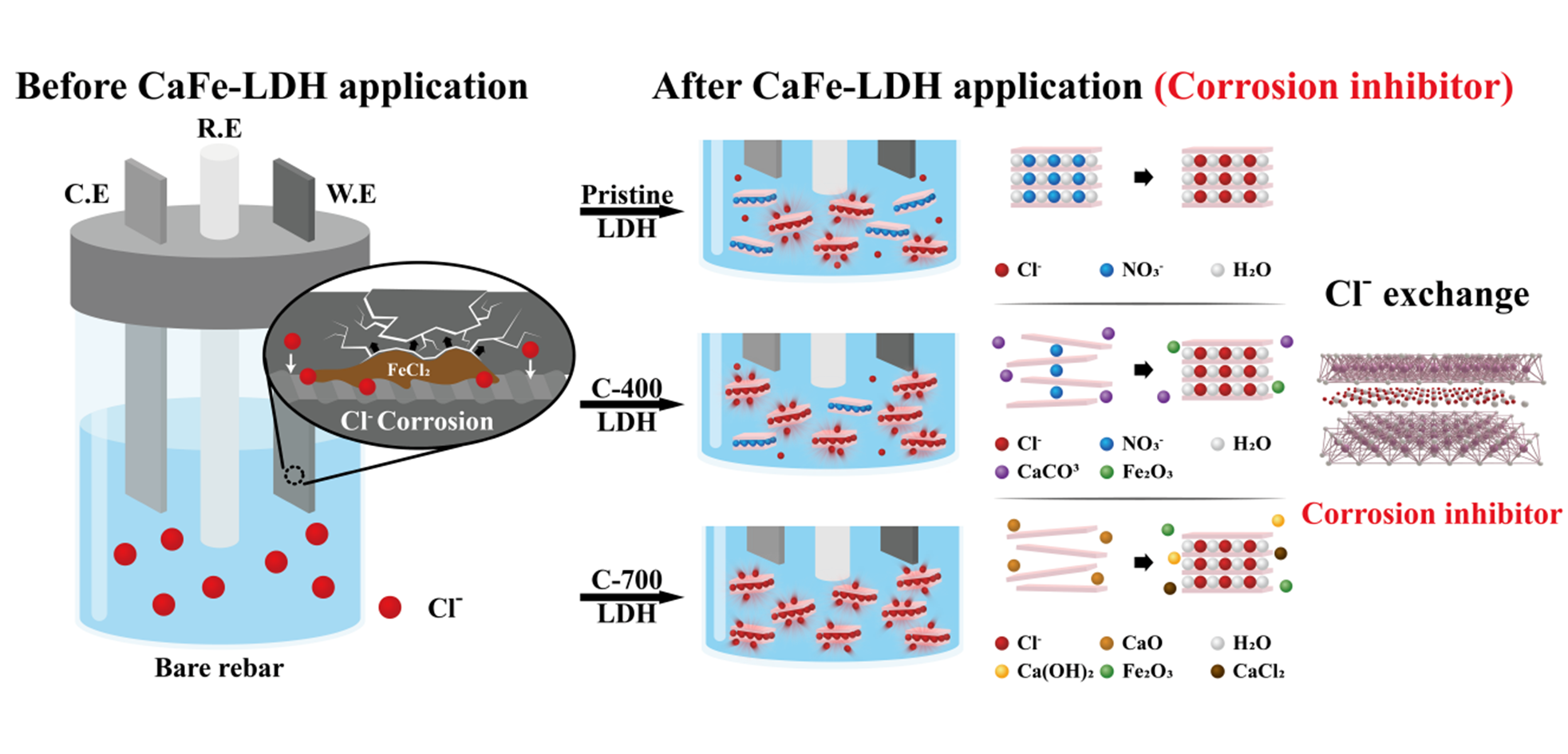

Supplement: Supplementary file 1 [file Image1.TIF]
