# Supplementary material for: CaFe-Based Layered Double Oxides With Superior Iron Alloy Corrosion Inhibition Behaviors in Aggressive Seawater Environment
Source: Front Chem. 2022 Feb 7;10:813008. doi: 10.3389/fchem.2022.813008 (PMC8858811; doi:10.3389/fchem.2022.813008)

**Supporting Figure captions**

**SI. 1.** XRD patterns of CaFe-LDHs and LDOs prepared with a variation in (a) molar ratio of Ca^2+^:Fe^3+^ and (b) pH (fixed molar ratio of Ca^2+^:Fe^3+^ =2:1)

**SI. 2.** XRD pattern of CaFe-LDOs calcined at 400 ℃ in air and Ar.

**SI. 3.** TGA curve obtained for calcium carbonate in air.

**SI. 4.** FT-IR spectra of CaFe-LDHs and LDOs showing different structural transformations according to the thermal treatment conditions: (a) View of overall range and magnified views in the ranges of (b) 1200~1700 cm^-1^, (c) 2500~3900 cm^-1^, and (d) 500~1000 cm^-1^.

**SI. 5.** FT-IR spectra of pristine LDH, C-400 LDOs and C-700 LDOs after chloride adsorption.

**SI. 6.** SEM images acquired before anion adsorption for (a) pristine LDHs, (b) C-400 LDOs, (C) C-700 LDOs and after anion adsorption for (a’) pristine LDHs, (b’) C-400 LDOs and (C’) C-700 LDOs.

**List of SI**

**SI. 1.**


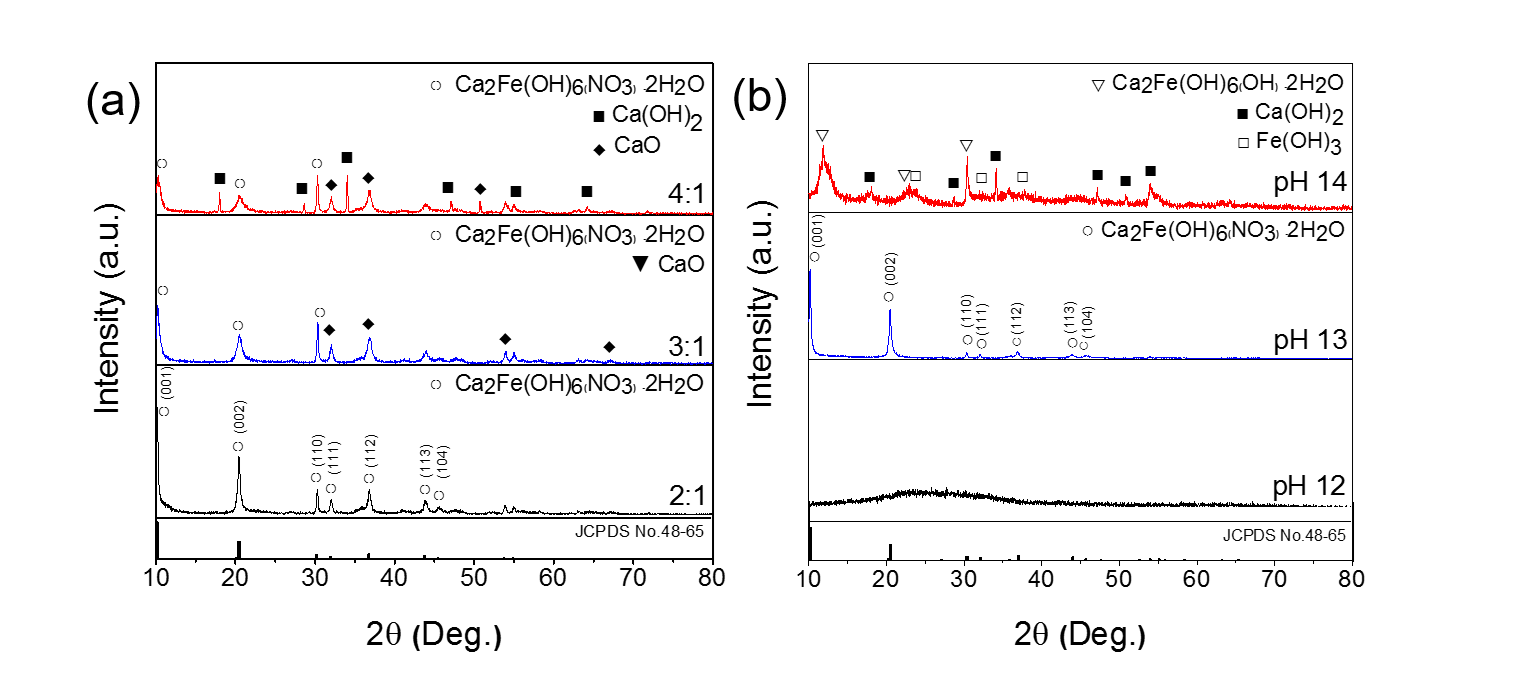


**SI. 2.**

**
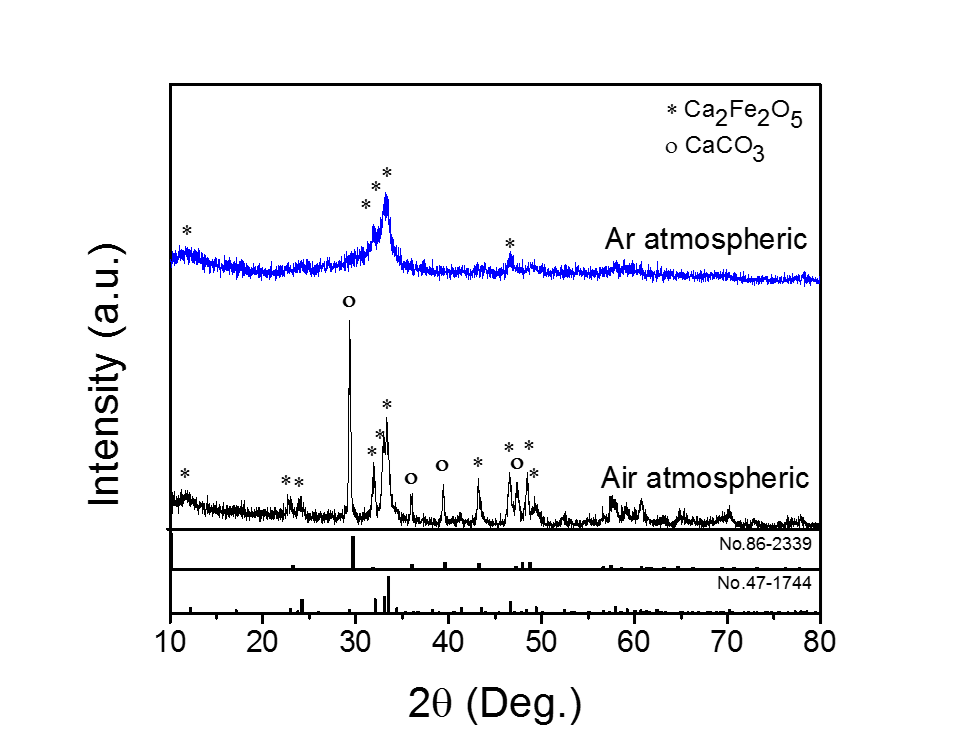
**

**SI. 3.**


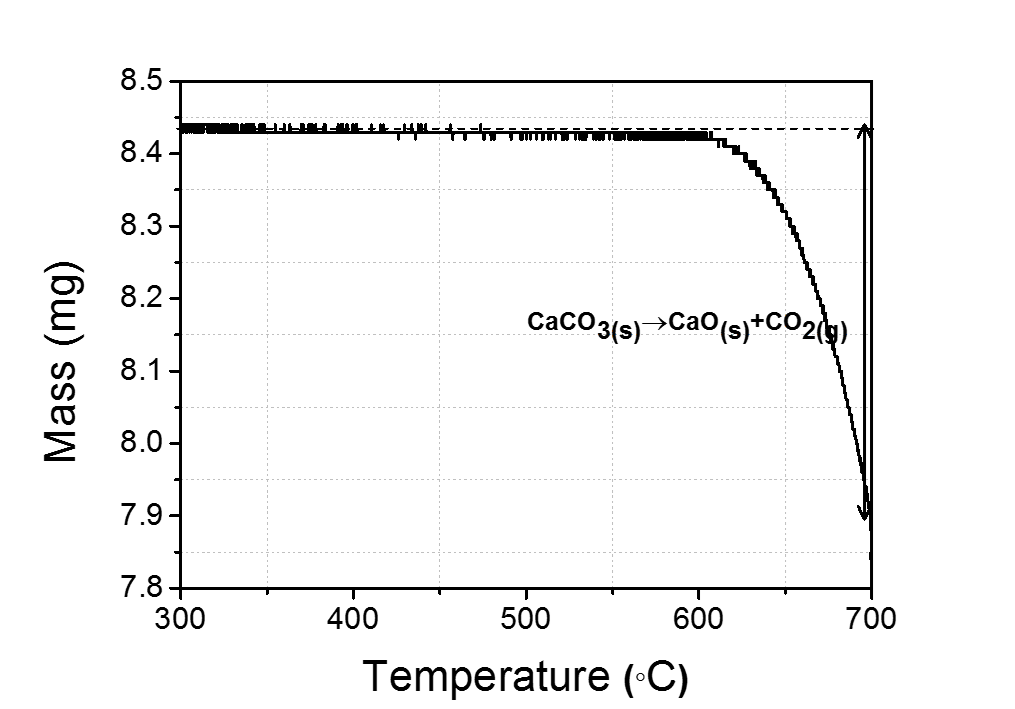


**SI. 4.**


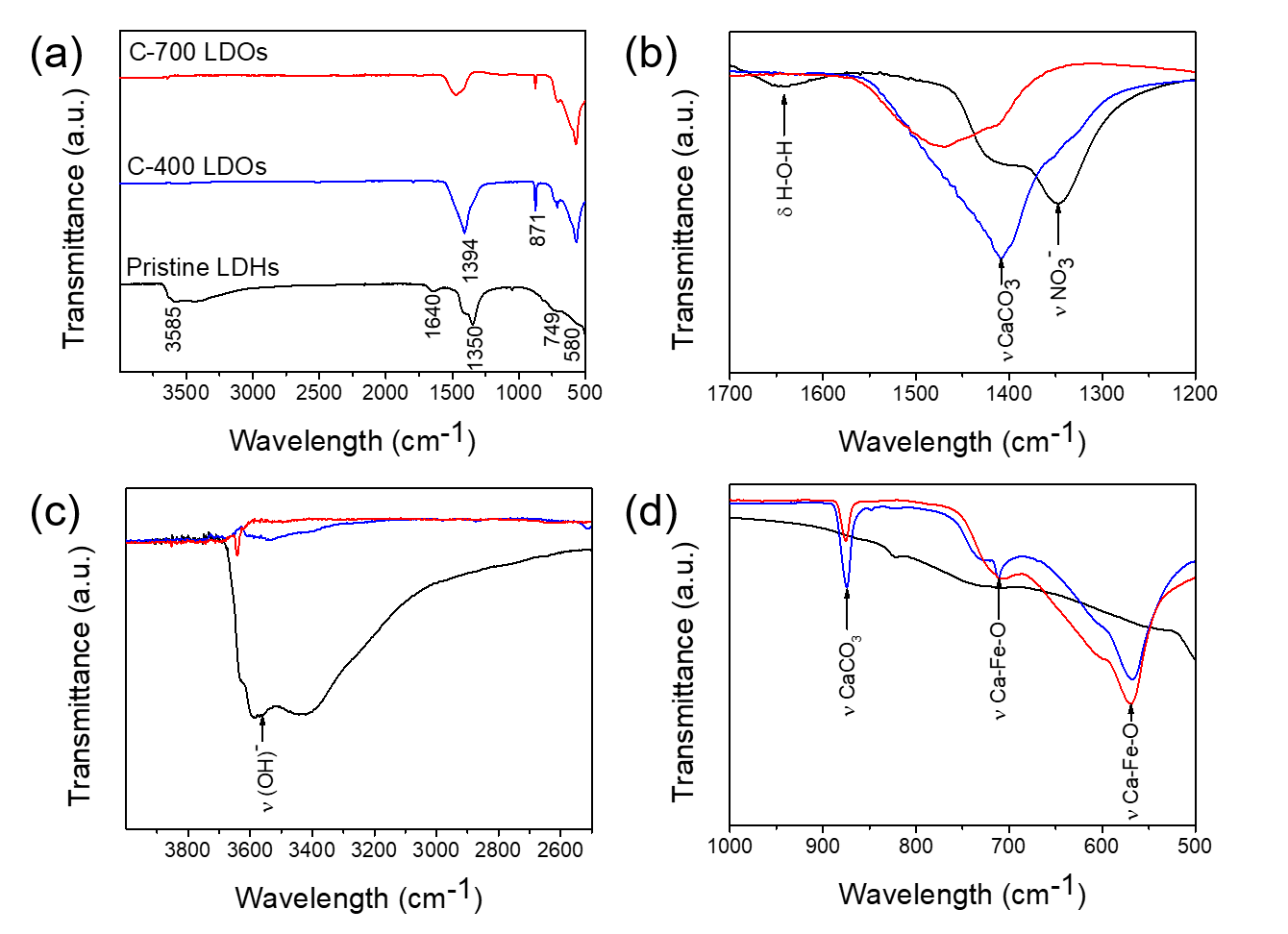


**SI. 5.**


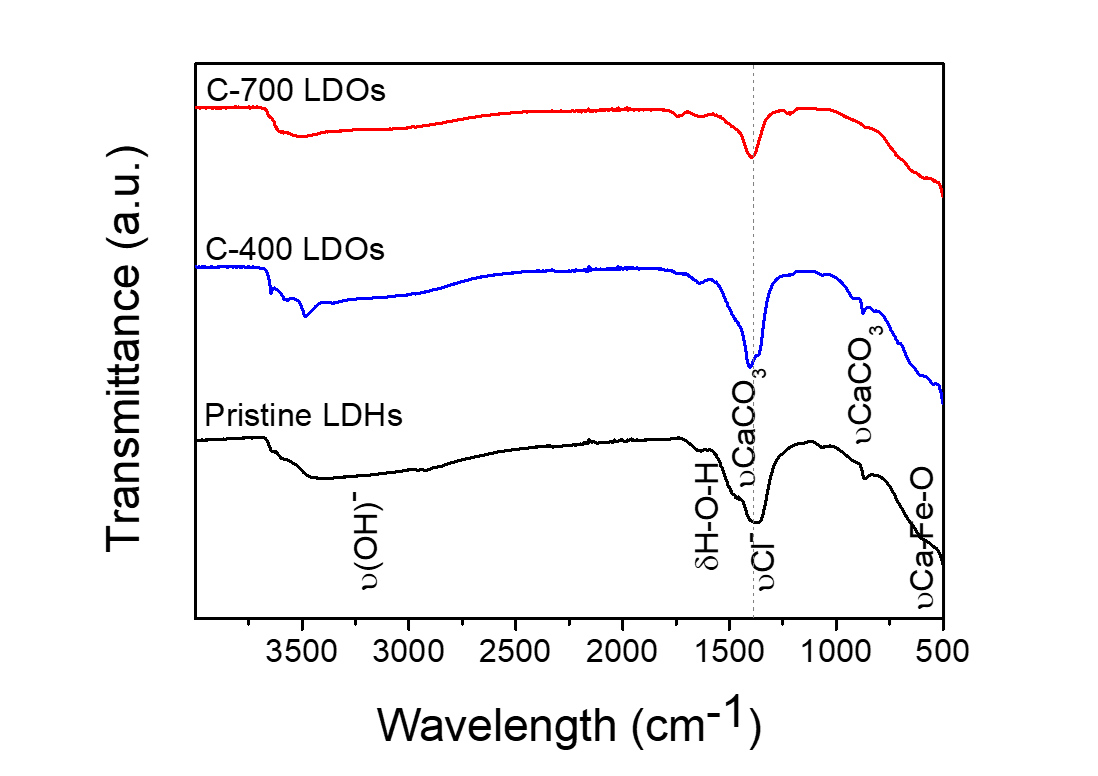


**SI. 6.**


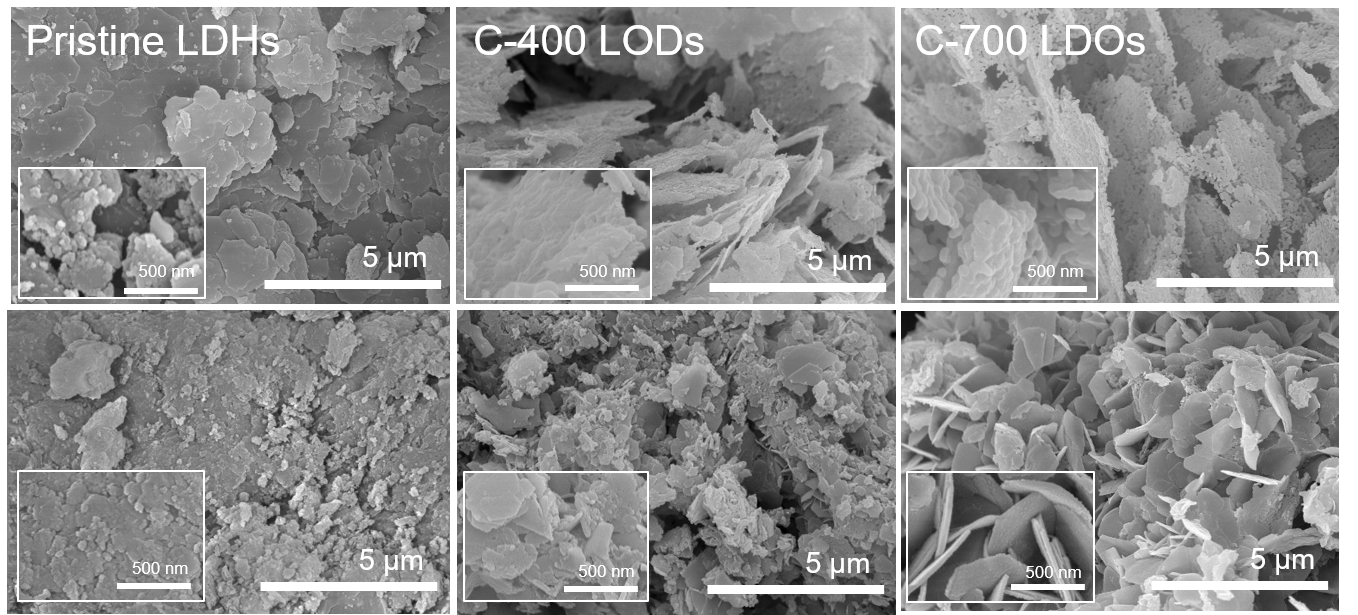

Supplement: Supplementary file 2 [file DataSheet1.docx]
